# Psychometric Properties of the Spontaneity Assessment Inventory-Revised (SAI-R): The 3-Factor Hypothesis

**DOI:** 10.3390/ijerph19137924

**Published:** 2022-06-28

**Authors:** Paulo Martins, António-José Gonzalez, Margarida Pedroso de Lima, António Rosado

**Affiliations:** 1 Laboratory of Sport Psychology, CIPER, Faculdade de Motricidade Humana, Universidade de Lisboa, Estrada da Costa, 1499-688 Cruz-Quebrada, Portugal; arosado@fmh.ulisboa.pt; 2 Applied Psychology Research Center Capabilities & Inclusion, ISPA—University Institute of Psychological, Social and Life Sciences, 1149-041 Lisbon, Portugal; gonzalez@ispa.pt; 3 Department of Clinical Psychology, Faculty of Psychology and Educational Sciences, University of Coimbra, 3004-531 Coimbra, Portugal; mplima@fpce.uc.pt

**Keywords:** psychodrama, spontaneity, psychological tests

## Abstract

Spontaneity and its connections to mental health and wellbeing are a central issue in both theory and practice of psychodrama, and the specialized literature sometimes even associates the presence of pathology to a lack of spontaneity. This paper describes spontaneity in Moreno’s theory, its assessment, its association with other measures and concepts, and scientific advances in the field. Specifically, we present results obtained with the English and Portuguese-language versions concerning its assessment. The main purpose of this work was to study the spontaneity measure (SAI-R) in a 3 first-order factors version for a Portuguese sample of 439 subjects, divided into two independent samples. The mean age of participants was 25.6 years (SD = 10.2), and ages ranged between 18 and 64 years. Data was collected through an online platform of a Portuguese market research company. The results revealed that the 3-factor model has acceptable validity for items 1 (“Creative”) and 7 (“Euphoric”), confirming the composite reliability, the convergent validity, but not the discriminant validity. Through multi-group analysis, the model proved to be stable. Notwithstanding the need for complementary studies, including clinical samples, the SAI-R is a short and valid instrument in clinical and non-clinical contexts when evaluating spontaneity.

## 1. Introduction

Spontaneity is one of the key concepts in psychodrama theory, and it is considered one of the focuses for psycho-dramatists to guide their daily clinical practice. Not only is clients’ spontaneity seen as an outcome of psychodrama and a desirable state connected to several health-related variables [1,2], but the therapists’ spontaneity is highly valued too [3]. These aspects make the creation and development of spontaneity assessment instruments a clear goal for the psychodrama community.

In one of his most influential works, “Who shall survive?”, J.L. Moreno [1] defined spontaneity as an appropriate response to a situation or a new response to an old situation. He suggested that the answer to the question in the book’s title lies in spontaneity itself. More specifically, the survivors will be spontaneous and creative personalities. He conceived it as non-accumulative energy that needs to be used at the moment, in the here-and-now. Assessing it was an important issue for the development of spontaneity research.

J.L. Moreno himself left several writings about spontaneity assessment, suggesting proceedings that demand an adequate setting, the presence of those to be evaluated, and a set of (more or less) standardized situations to be dramatized. These situations would allow the observation of the behavioral responses of the subject, as well as their evaluation in terms of adequacy, originality, and speed [4] (p. 151) [2] (p. 128). This evaluation form allowed Moreno to systematically study spontaneity-connected phenomena and develop his theories about them. Nevertheless, these proceedings are not only not economical in terms of time and number of evaluators needed but also lack some characteristics associated with psychometric tests, such as standardization of conditions and evaluation criteria.

In an attempt to build a different epistemological and methodological perspective on Morenian spontaneity, the psychodrama and research community made efforts to develop psychometric tests to assess it (PAS: Personal Attitude Scale, [5]; PAS-II, [6]; SAI: Spontaneity Assessment Inventory, [2,7]; APTS: Adult Playfulness Trait Scale [8], with a spontaneity sub-scale). The Spontaneity Assessment Inventory was developed during the first decade of the 21st century by David Kipper from Roosevelt University, Chicago, and his collaborators [9]. Several developments led to its revised version, SAI-R [2,9,10], which became the most commonly used instrument by the psychodrama community, since it was initially inspired by Moreno’s proposals and definitions of spontaneity.

With the first version of this instrument, several correlational studies were developed to confirm some of Moreno’s thesis about spontaneity. Kipper and Hundal [9] found significant positive relations between SAI scores and all of Friedman’s Well-being scales. Christoforou and Kipper [10] found, in a sample of 85 university students, significant negative correlations with scores on both state and trait anxiety scales and with scores on a scale measuring Obsessive-Compulsive symptoms. Using a Temporal Orientation scale, they also found a significant positive correlation between spontaneity scores and the Orientation to Present subscale (non-significant correlations were found with Past and Future Orientations). All these findings can easily be connected with Moreno’s suggestions that spontaneity is negatively connected to anxiety and obsessive-compulsive disorders and that spontaneous persons are more connected with the here-and-now.

In possession of the revised version of the spontaneity inventory, Kipper and Shemer [2] confirmed the positive correlations with well-being scales and found negative correlations with perceived stress, while Davelaar et al. [7] found positive correlations with intrinsic (but not extrinsic) motivation, perceived self-efficacy, and self-esteem. These results are compatible with most of Moreno’s conceptualization of spontaneity as energy connected with health in general, an ability to behave in a free form but within the boundaries of adequate responses. The statement that spontaneity is not connected to impulsivity and that this characteristic is more a case of spontaneity pathology is supported by Kipper, Green, and Prorak [11], who found a significant negative correlation between scores on tests measuring these concepts.

Recently, a paper from Kipper and Beasley [12] explored the presence of three different dimensions in SAI-R. In this work, the authors performed a Factor Analysis and, based on the statistical data and the psychodrama theory, proposed a tridimensional perspective for the 18 items of the inventory. The first and most important factor, including eight items and explaining 35% of the variance, is connected to Vigor and Pleasure. This could easily relate to Moreno’s characterization of spontaneity as energy [4], connected to “satisfying positive feelings” [11] (p. 41) and its connections with health and well-being [2].

The other two factors are smaller, comprising five items each and explaining only 6.8 and 3.5% of the variance, respectively. The authors connect Factor 2, named Freedom, and including items like “Free to invent” or “Creative”, to “a sense of freedom of actions and expressions and an attitude of being unhindered by conventional boundaries” [12] (p. 9).

The last, smaller factor, is nevertheless connected to a critical feature in Moreno’s proposals about spontaneity, clearly distinguishing it from impulsivity (as seen above). Focus and Control are the main characteristics of this third factor. Several authors [11,13] have suggested the proximity between Moreno’s spontaneity and flow states, as proposed by Csikszentmihalyi [14], both being considered “optimal experiences”.

When performing their exploratory analysis, Kipper and Beasley [12] found an alternative 2-factor model, but they made a theoretically based decision to choose the 3-factor model because it was more adequate to the Morenian theory that suggests spontaneity is a pleasurable energy connected to free will acts and deep involvement in tasks. Nevertheless, they finish their paper with two concerns that might hinder the validity of the work: the participants in their sample were mainly females (76%), and all of them were university students.

More recently, in Portugal, there was an SAI-R validation keeping the unidimensional proposal but suggesting the call for theoretical meaning of the values that overlap and with lower loadings, which might imply reviewing the items and exploring other factors as stated by Kipper and Beasley [15].

Furthermore, literature in expressive arts in general, and particularly in psychodrama, points out the need for further studies, not only correlational but also randomized clinical trials [16], for which valid psychometric instruments are needed. Finally, several authors [12,17] suggested studies that would bring SAI-R to different cultural contexts. This being said, our work aims to address not only these concerns, making SAI-R a stronger and more valid instrument, but to allow the development of a theoretical model that connects spontaneity and mental health variables [18]. As stated above, evidence shows that higher levels of self-perceived spontaneity and creative adaptation are strongly connected, suggesting that spontaneity could be seen as a cornerstone for coping with life’s challenges and critical events, as, for instance, the recent demands connected to the COVID-19 pandemic situation [19,20].

Finally, SAI-R is the most used spontaneity test in the international psychodrama community, making access to a robust Portuguese version an important goal. For these reasons, this study aims to further validate SAI-R in its Portuguese version and test its tri-dimensionality, offering the community of researchers, psychologists, clinicians, and psycho-dramatists the possibility of using a valid instrument for diagnostic and therapeutic efficacy in their interventions.

## 2. Materials and Methods

### 2.1. Participants and Study Design

This study included 439 participants between the ages of 18 and 64 years, with a mean age of 25.6 years (SD = 10.2 years). Among these, 303 were women (69%), and 136 were men (31%). The survey was carried out in several regions of Portugal, but most (77.9%) of the participants were from Lisbon. The majority (*n* = 368, 85%) have a high school education (*n* = 244, 55.6%) or a University degree (*n* = 124, 28.2%). More than half of the participants were students (*n* = 288, 65.6%). Methodologically we used a research design to verify the reliability and validity of the Portuguese version of the Spontaneity Assessment Inventory-Revised translated into Portuguese so that it can be applied in Portuguese-speaking contexts.

### 2.2. Instrument

We used the 3-factors SAI-R proposed by Kipper and Beasley [12], which comprises eighteen items distributed in three dimensions, namely: Vigor/Pleasure, Freedom, and Involvement/Focus (see Table 1). As with the one-dimensional SAI-Rp, subjects must indicate feelings and thoughts that people experience in different situations during a typical day. Moreover, similarly to the original scale, responses are given on a 5-point Likert-type scale ranging from 1 = Very Weak (never/rarely) to 5 = Very Strong (often/always). Sample items include, for example: “1. Creative; 2. Happy; 8. Free to act, even overreact; 9. Living fully with balance; 17. Able to do anything, within limits; 18. Cheerful.” As for the overall reliability, we found a Cronbach’s alpha of 0.92, a value that can be considered excellent. As for factor reliability, Cronbach’s alpha ranged from 0.71 to 0.88, thus considered as good to very good [12].

### 2.3. Procedures

Data collection was performed online by a Portuguese market research company over a 15 day period. Upon selecting and meeting with the aforementioned company, we outlined our goal of validating this scale for the Portuguese population, as well as the need to gather data on a wide sample, capable of representing the Portuguese population in the best possible manner. At the end of this 15 day data-gathering period, we received the resulting data in one single moment. As for the procedure of data collection, before completion, all participants were instructed about the study’s goals and the importance of their participation, being asked at the end to mark/accept the informed consent form. The study’s importance and participation were emphasized, and all subjects agreed to participate voluntarily, with guaranteed anonymity and confidentiality of responses. In the follow-up, explanations were also given about the mode of response. Questionnaires were self-administered, and the completion took approximately 12 min.

### 2.4. Statistical Analysis

To analyze the data, we used structural equation modeling (SEM) available on AMOS 27.0 software (SPSS Inc, Chicago, IL, USA). To assess the factorial validity of the SAI-Rp, we performed a confirmatory factor analysis as described by Byrne [21,22]. For that, we used the maximum likelihood method [23]. To verify the normality and the inexistence of outliers, we used the skewness and kurtosis coefficients and the Mahalanobis distance (D^2^), respectively [23]. To assess the adequacy of the model, we used the chi-square statistic (χ^2^); the Ratio of chi-square by degrees of freedom (χ^2^/gl), considering that χ^2^/df with a value less than 5.0 indicates an acceptable fit of the model to the data, and a value less than 3.0 indicates a good fit of the model to the data [23,24]. Then the CFI (Comparative Fit Index), GFI (Goodness Fit Index), and TLI (Tucker-Lewis Index) indices were tested. Values greater than 0.90 and greater than 0.95 indicate good and very good fit, respectively [23]; CFI (Comparative of Fit Index) GFI (Goodness of Fit Index) and TLI (Tucker-Lewis Index) indices, where values ranging from 0.60 to 0.80 indicate good fit and values greater than 0.80 indicate very good fit, were also tested. Finally, RMSEA (Root Mean Square Error of Approximation) was checked. When this index is less than 0.06 with a probability *p* (rmsea ≤ 0.05) not significant, it indicates a good fit of the model [22]. The significance of the structural weights was assessed using the *Z*-tests produced by AMOS for structural model fit and to test the relationships between the constructs, considering Z ≥ 1.96 and statistical significance when *p* ≤ 0.05 [25]. To assess the consistency of the factors, we calculated both the Cronbach alpha and the composite reliability [26], while to assess composite validity, we calculated the average variance extracted (AVE) values [27]. Cronbach alpha values equal to or greater than 0.70 are considered good, and composite reliability equal to or greater than 0.70, and AVE equal to or greater than 0.50 are indicators of reliable and valid constructs [27]. Discriminant validity was established when AVE for each construct exceeded the squared correlations between that construct and the remaining ones [27]. In addition, we performed a multi-group analysis to compare the first sample with a second one, thus evaluating cross validity. The model’s invariance in both samples was verified by comparing the unconstrained model with constrained models (factor loadings fixed and variances/co-variances fixed). According to the χ^2^ statistic [28], factorial invariance was accepted when the models did not differ significantly (*p* > 0.05).

## 3. Results

The results do not confirm a normal distribution (see Table 2), considering that skewness and kurtosis values are below 3 and 7, respectively [24]. Also, the Mardia coefficient (Coefficient = 69.75) indicated no multivariate distribution [29]. Finally, the Kolmogorov-Smirnov test did not reveal the normality of the sample distribution [K-S (439) = 0.085, *p* = 0.001)]. Thus, we used Bollen and Stine’s bootstrapping (B-S) [14] procedure to adjust the *p*-value of the chi-square statistic. The overall assessment of the structural model (χ^2^ (132) = 552.474, B-S *p* < 0.001; χ^2^/df = 4.185; CFI = 0.89; GFI = 0.87; TLI = 0.87; RMSEA = 0.085; 90% CI [0.078–0.093]) indicates an acceptable fit of the model to the data. The values of CFI, GFI, and TLI meet the recommended criterion (>0.80) for acceptable fit [14]. Despite this, not all estimated factor loadings met the recommended cutoff point of 0.50 [28]. Thus, it was decided to remove item 1 (“Creative”) and item 7 (“Euphoric”) since they have a factor loading of 0.41 and 0.40, respectively. Further, the RMSEA showed an unacceptable fit [25,26].

In the version after the removal item 1 (“Creative”) and item 7 (“Euphoric”) (see Table 3), the overall assessment of the structural model (χ^2^ (101) = 338.194, B-S *p* < 0.001; χ^2^/df = 3.348; CFI = 0.93; GFI = 0.91; TLI = 0.92; RMSEA = 0.07; 90% CI [0.065–0.082]) indicates an acceptable model fit to the data. The values of CFI, GFI, and TLI meet the recommended criterion (>0.90) for good fit [26]. RMSEA also showed an acceptable fit [25,26]. Again, it should be noted that in factor 2 (“Freedom”), not all items showed adequate factor loading (≧0.50), namely item 3 (“Uninhibited”) and item 8 (“Free to act, even outrageously), nor adequate individual reliability (R2 ≧ 0.25), scoring 0.20 and 0.22 respectively. Notwithstanding, the remaining items displayed adequate individual reliability ranging between 0.41 and 0.70. The Z-test values also indicated statistical significance ranging between 11.71 and 14.21 [26]. Composite reliability of each factor was achieved, with values ranging between 0.83 and 0.88, far exceeding the recommended minimum of 0.60 [30]. Evidence of convergent validity was also confirmed since the AVE values were above 0.50, which is the recommended cut-off value for this index [27,31]. Additionally, the total scale obtained a mean of 59.30 (SD = 11.25), and the mean and standard deviation values by gender were 55.59 (10.79) for women and 59.07 (10.50) for men and this difference was not significant.

Additionally, a Cronbach’s alpha of 0.92 was obtained, a value that may be considered excellent for the reliability of the overall instrument. Concerning the reliability of the subscales, the Vigor/Pleasure subscale obtained Cronbach’s alpha of 0.88, which is considered an indicator of very good reliability, the Freedom subscale obtained Cronbach’s alpha of 0.71, indicating good reliability, and the Involvement/Focus subscale obtained Cronbach’s alpha of 0.83, thus qualifying for very good reliability.

**Figure 1:**
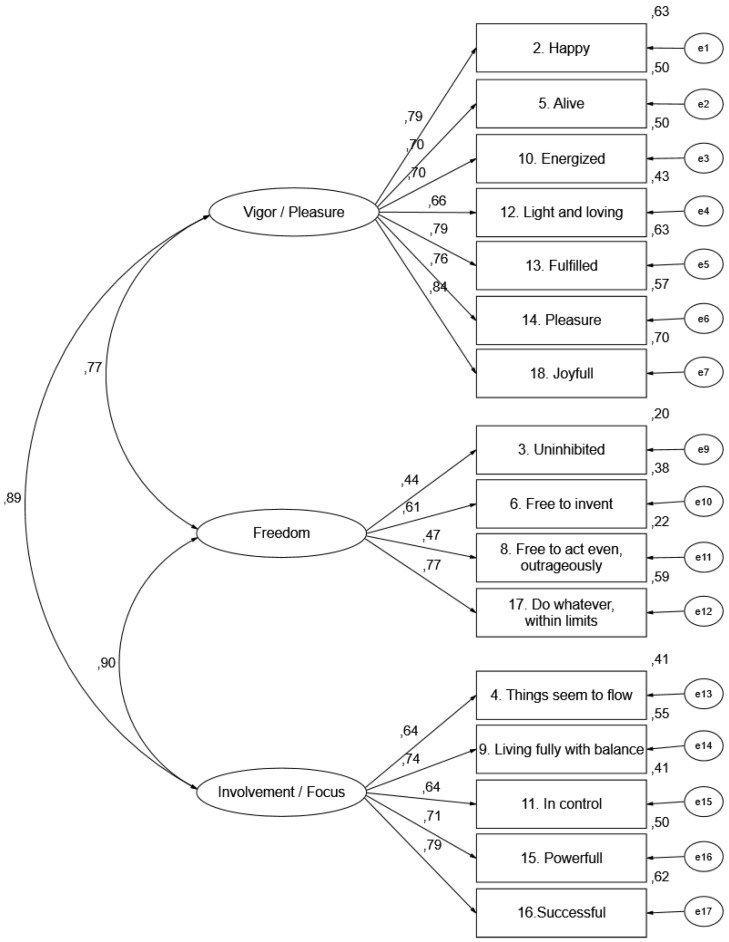
CFA re-specified of the 3-first-order factors of the SAI-Rp.

The communalities were also satisfactory since, although several items had low values, on average, the cutoff point of 0.40 was reached. Figure 1 shows the re-specified 3-factor with 16 items SAI-R model.

The average variance extracted and the squared correlation of the factors are presented in Table 4. The evidence of discriminant validity was not accepted since the squared correlation of factor 3 (Involvement/Focus) and factor 1 (Vigor/Pleasure) exceeded the AVE values.

### Cross Validity

The stability of the model was also studied by checking cross validity (Table 5). Specifically, a multi-group analysis was performed on two equivalent samples. (Women × Men) (Sample 1 = 303; Sample 2 = 136). As shown in Table 5, the fit of the free model (Model 1: χ^2^ (230) = 508.93; PCFI = 0.78; PGFI = 0.67; CFI = 0.92; GFI = 0.88; RMSEA = 0.05) proved to be acceptable [25].

Similarly, the models with fixed variances (Model 2: χ^2^ (244) = 522.13 (B-S *p* < 0.51); PCFI = 0.83; PGFI = 0.70; GFI = 0.87; CFI = 0.92; RMSEA = 0.05 ₩ and the model with fixed residuals (Model 3: χ^2^ (250) = 532.77 (B-S *p* <0.23); PCFI = 0.85; PGFI = 0.71; GFI = 0.87; CFI = 0.92; RMSEA = 0.05), also showed an acceptable fit. The χ^2^ statistic showed no significant differences between Model 1 and Model 2 (χ^2^dif (14) = 13.2; B-S *p* = 0.51) or Model 1 and Model 3 (χ^2^dif (20) = 23.8; B-S *p* = 0.23). Thus, the results demonstrated model invariance in both samples, showing that the factor structure of SAI-Rp is stable in two independent samples and, to that extent, indicating cross validity [25,28].

## 4. Discussion

The purpose of this work was to study the characteristics of the Portuguese version of the spontaneity measure (SAI-R) in a 3-first-order factors version (Vigor/Pleasure; Freedom and Involvement; Focus), following the suggestions of Kipper and Beasley [12]. Their proposal seems to provide clues that describe feelings and thoughts corresponding to first-order factors about the state of spontaneity and that these would be more in line with Moreno’s thinking. The authors propose that, according to Moreno [4], spontaneity is an innate, pleasurable drive or energy. It is characterized by deep involvement in the task, accompanied by a sense of freedom. Complementarily, the authors, referring to the etymology of the word spontaneity, connect it to free will. It has a directional sense that aims to trigger the creative process. Finally, they suggest that the three-factor solution (Vigor/Pleasure, Freedom, and Involvement/Focus) captures the essence of spontaneity as characterized by these ideas.

In our study, SAI-R emerges as a short, versatile, and valid instrument when one intends to evaluate spontaneity. As found in previous studies, both with the English [7]) and non-English versions ([15,32,33,34] SAI-R showed overall good reliability (with Cronbach’s alphas not lower than 0.81 and typically above 0.90), and we could confirm that in the present work. Furthermore, both composite and convergent reliabilities were found.

We used the unidimensional SAI-R translated to Portuguese by Gonzalez et al. [15]. However, because overlaps were found between items 1—“Creative” and 6—“Free to invent”, a discussion is needed about how to deal with this information. These items were translated to Portuguese literally in the first case (“Criativo”). However, both the translators and the specialists agreed that in the case of “Free to invent”, the direct translation (“Livre para inventar”) has an ambiguous interpretation in Portuguese that could connect this item to a somehow inadequate activity once it can be interpreted as “free to do anything”. So, the translation option was to use “Free to create”, thus explaining the overlap with item 1. Because the model displays better fit indexes in the absence of item 1 (“Creative”), we suggest its withdrawal from the inventory. Another item that we suggest withdrawing is item 7 (“Exhilarated”). This suggestion is due to two reasons. First, this item shows poor factor loading (below-recommended cut-off point) [35]. Secondly, there are no error co-variances in the factor that can be correlated to improve the adequacy. Additionally, our findings align with Moreno’s theoretical assumption that spontaneity is not connected to impulsivity.

Finally, the modification indices (MI) suggested the error correlations between items 3—“Uninhibited” and 17—“Do whatever within limits” (MI = 4.146, EPC = −0.60), but it was not confirmed that the two errors, when correlated, significantly improve the model fit. More to the point, examining the content of these items made clear that they do not have any degree of redundancy, and so it was decided to keep the model without these correlated items. As said, in Portugal, there was a unidimensional validation of the SAI-R [15], and this study found the same overlaps we have indicated. However, a more conservative stance was taken, as the authors decided to keep all items and stay close to the original version’s theoretical position. The authors also suggested evaluating the SAI-R to inspect the correlation between Vigor/Pleasure, Freedom, and Involvement/Focus to identify factors that influence spontaneous behavior, as stated by Kipper and Beasley [12]. Following these suggestions, we found some support for this hypothesis since our study revealed that the proposed 3-factor structure with 16 items showed both construct and content validities, as well as good scale reliability. Confirmatory factor analysis revealed an acceptable fit of the model to the data. Composite reliability and convergent validity were also confirmed [22]. Moreover, although discriminant validity was not achieved in factor 3 (“Involvement/Focus”), we consider that the inventory is a reliable tool to measure spontaneity.

Finally, this validation is also an important and necessary step for studies done by the Portuguese language spoken community of psycho-dramatists and for researchers interested in the subject of spontaneity as a precursor of creativity in general. This 3-factor SAI-Rp is a useful tool to assess the levels of spontaneity in various contexts. It contributes to the urgent call to enhance scientific therapeutic procedures, especially in the case of Psychodrama, a psychotherapy model whose theory places spontaneity as a central variable in the promotion of mental health.

## 5. Conclusions

This study validated the 3-factor SAI-R scale proposed by Kipper and Beasley [12] for the Portuguese context and assessed its validity and reliability. The original 3-factor inventory consisted of 3 specific factors (i.e., Vigor/Pleasure with eight items; Freedom with five items; Involvement/Focus with five items) distributed over 18 items. As a result of the confirmatory factor analysis, the Vigor/Pleasure scale was reduced to seven items, and the Freedom scale was reduced to four items, while the Involvement scale remained with five items. Hence, we obtained a 16 item scale. For a better clarification of the translation and adaptation of the scale, we included the final Portuguese version of the scale as Appendix A.

Moreover, the use of the measure of spontaneity benefits psychodrama theory itself since its use may be an opportunity to reinvent it by meeting the new demands coming from the contexts where it is used [18]. On the other hand, in the evaluation of the therapeutic process, the use of the measure of spontaneity may help innovate psychodrama-based interventions [17]. Finally, with the measure now presented, it is possible to correlate spontaneity with creative adaptability in Portuguese-speaking contexts and compare it with other cultural contexts, for example, in the case of the pandemic outbreak of COVID-19 [20,36].

## 6. Limitations and Future Research

As any other research, this study includes some limitations. First, the participants are in the majority students, which limits to some degree the generalization of the results. Therefore, future studies ought to focus on populations with more distributed traits. Second, due to both a certain overlap of items and the sample collection procedure having taken place in a non-clinical setting (which limits to a certain degree the accuracy attained), cautious use of this tool is recommended when assessing levels of spontaneity. Doing so will allow for the analysis of the impact of the interventions within the contexts that differ from the one analyzed in this study. Additionally, few studies have, up until now, focused on spontaneity-based interventions. Of the few studies available, none has analyzed the results concerning the three dimension of SAI-R, and so, it would be interesting to see the continuation of such studies taking place.

## Figures and Tables

**Table 1:** Dimensions, description, and corresponding items from the original 3-factor version of the SAI-R (Kipper, & Beasley, 2016).

Factors	Description	Items
Vigor/Pleasure	Reflects spontaneity as a pleasantly exciting experience and that it is forceful.	2, 5, 7, 10, 12, 13, 14, 18
Freedom	Reflects spontaneity as a pleasurable experience; it is also characterized by a sense of freedom of actions and expressions and an attitude of being unhindered by conventional boundaries.	1, 3, 6, 8, 17
Involvement/Focus	Reflects spontaneity as a focused and deliberate state of mind as opposed to impulsivity, a form of expression marked by emotional, unfocused, and uncontrolled out-busts typically associated often with unpleasant feelings.	4, 9, 11, 15, 16

**Table 2:** Descriptive statistic of the 3-factors SAI-Rp.

Item	M	SD	Min	Max	Ass.	Kurt.
**Vigor/Pleasure**						
2. Happy	3.95	0.96	1	5	−0.882	0.591
5. Alive	2.64	0.94	1	5	0.003	−0.375
7. Exhilarated	3.39	0.97	1	5	−0.492	−0.004
10. Energized	3.30	0.99	1	5	−0.368	−0.263
12. Light and loving	3.21	1.09	1	5	−0.429	−0.566
13. Fulfilled	3.46	0.88	1	5	−0.420	−0.037
14. Pleasure	3.75	0.94	1	5	−0.666	0.239
18. Joyful	3.21	0.83	1	5	−0.736	0.776
**Freedom**						
1. Creative	3.21	0.97	1	5	−0.293	−0.161
3. Uninhibited	3.10	0.95	1	5	−0.052	−0.183
6. Free to invent	3.32	0.98	1	5	−0.240	−0.355
8. Free to act, even outrageously	2.77	0.97	1	5	0.022	−0.321
17. Do whatever, within limits	3.47	0.98	1	5	−0.443	−0.152
**Involvement/Focus**						
4. Things seem to flow	3.32	0.80	1	5	−0.407	0.300
9. Living fully with balance	3.21	1.05	1	5	−0.281	−0.507
11. In control	3.48	0.92	1	5	−0.426	0.040
15. Powerful	2.92	1.01	1	5	−0.161	−0.517
16. Successful	3.15	0.98	1	5	−0.325	−0.235

Notes: M = mean; SD = Standard deviation; Min = minimum; Max = maximum; Ass. = Asymmetry; Kurt. = Kurtosis.

**Table 3:** Factor loadings, Z-values, Communalities, Cronbach alpha and Composite reliability of the SAI-Rp model at 3 factors with 16 items.

Factors/Items	λ	*Z*-Value	C	α	CR
**Vigor/Pleasure**				0.88	0.90
2. Happy	0.79	12.67 ***	0.61		
5. Alive	0.70	13.59 ***	0.45		
10. Energized	0.70	13.57 ***	0.53		
12. Light and loving	0.66	13.89 ***	0.41		
13. Fulfilled	0.79	12.84 ***	0.58		
14. Pleasure	0.76	13.16 ***	0.52		
18. Joyful	0.84	11.79 ***	0.66		
**Freedom**				0.71	0.67
3. Uninhibited	0.44	14.03 ***	0.14		
6. Free to invent	0.61	12.31 ***	0.41		
8. Free to act, even outrageously	0.47	13.80 ***	0.23		
17. Do whatever, within limits	0.77	10.39 ***	0.25		
**Involvement/Focus**				0.79	0.86
4. Things seem to flow	0.64	13.65 ***	0.32		
9. Living fully with balance	0.74	12.82 ***	0.47		
11. In control	0.64	13.70 ***	0.39		
15. Powerful	0.71	13.14 ***	0.43		
16. Successful	0.98	12.13 ***	0.52		

Notes: λ = factor loading; Z-value = Critical ratio; C = Communalities; α = Cronbach alpha; CF = Composite reliability; *** *p* < 0.001.

**Table 4:** Mean (M), standard deviation (SD), and squared factor correlations.

Factor	AVE	Correlations Matrix		
		1	2	3
1. Vigor/Pleasure	0.67	1.00		
2. Freedom	0.57	0.31 **	1.00	
3. Involvement/Focus	0.57	0.62 **	0.40 **	1.00

Note: ** *p* < 0.01.

**Table 5:** Results of the CFA Multi-Group Analysis of the SAI-Rp 3-Factor.

Models	χ^2^	gl	∆χ^2^	∆gl	B-S p	PCFI	PGFI	GFI	CFI	RMSEA	IC 90%
Model 1	508.93	230	---	---	---	0.78	0.66	0.88	0.92	0.05	[0.05, 0.06]
Model 2	522.13	244	13.2	14	0.51	0.83	0.70	0.87	0.92	0.05	[0.05, 0.06]
Model 3	532.77	250	23.8	20	0.23	0.85	0.71	0.87	0.92	0.05	[0.05, 0.06]

Notes: Sample 1: *n* = 303; Sample 2: *n* = 136.

## Data Availability

Not applicable.

## Appendix A. SAI-Rp

O inventário seguinte foi elaborado para ajudar a entender os sentimentos e pensamentos que as pessoas vivenciam em diferentes situações durante um dia típico. Gostaríamos que partilhasse connosco as suas experiências relativamente a esse aspeto.

Instruções: Os 18 diferentes sentimentos e pensamentos apresentados abaixo referem-se à questão em negrito. Deverão ser avaliados nas escalas ordenadas de 1 = Muito Fraco (nunca/raramente) até 5 = Muito Forte (frequentemente/sempre), que aparecem à direita de cada item. Por favor leia cada item cuidadosamente e assinale com um círculo ou cruz o número que melhor descreve a intensidade dos seus sentimentos ou pensamentos. É importante que não deixe em branco nenhum dos itens. Não há respostas certas ou erradas.

**Table:**

Muito Fraca	Fraca	Nem Hraca nem Forte	Forte	Muito Forte
1	2	3	4	5

Com que intensidade tem estes sentimentos ou pensamentos durante um dia típico?

**Table:**

01 Feliz	1	2	3	4	5
02 Desinibido/a	1	2	3	4	5
03 As coisas parecem fluir	1	2	3	4	5
04 Vivo/a	1	2	3	4	5
05 Livre para criar	1	2	3	4	5
06 Livre para agir, até exageradamente	1	2	3	4	5
07 Vivendo plenamente com equilíbrio	1	2	3	4	5
08 Com energia	1	2	3	4	5
09 Com controlo	1	2	3	4	5
10 Leve e amorosamente	1	2	3	4	5
11 Completo/a	1	2	3	4	5
12 Prazer	1	2	3	4	5
13 Poderoso/a	1	2	3	4	5
14 Bem sucedido/a	1	2	3	4	5
15 Capaz de fazer qualquer coisa, dentro dos limites	1	2	3	4	5
16 Alegre	1	2	3	4	5
